# Behavior and Food Consumption Pattern of the French Polynesian Population in the 1960s –1970s

**DOI:** 10.31557/APJCP.2019.20.12.3667

**Published:** 2019

**Authors:** Vladimir Drozdovitch, André Bouville, Tetuaura Tetuanui, Marc Taquet, Jacques Gardon, Constance Xhaard, Yan Ren, Françoise Doyon, Florent de Vathaire

**Affiliations:** 1 *Division of Cancer Epidemiology and Genetics, National Cancer Institute, NIH, DHHS, *; 2 *National Cancer Institute (retired) Bethesda, MD, USA, *; 3 *Research Institute for Development, Center IRD on Tahiti, Arue, Tahiti, French Polynesia, *; 4 *Hydrosciences Montpellier, Research Institute for Development, CNRS, University of Montpellier, Montpellier, *; 5 *National Institute for Health and Medical Research, Center for Research in Epidemiology and Population Health (CESP), INSERM U1018, Radiation Epidemiology Group, *; 6 *Institute Gustave Roussy, *; 7 *University Paris-Saclay, Villejuif, *; 8 *University of Lorraine, INSERM CIC 1433, Nancy CHRU, INSERM U1116, Nancy, France. *

**Keywords:** Behavior, food consumption, atmospheric nuclear weapons test, radiation, French Polynesia

## Abstract

**Background.:**

Reconstruction of radiation doses to the thyroid for a case-control study of thyroid cancer in French Polynesians exposed to radioactive fallout from atmospheric nuclear weapons tests during childhood and adolescence faced a major limitation on very little availability of information on lifestyle of French Polynesians in the 1960s–1970s.

**Method:**

We use the focus group discussion and key informant interview methodology to collect historical, for the 1960s–1970s, data on behavior and food consumption for French Polynesia population exposed to radioactive fallout from nuclear weapons tests conducted between 1966 and 1974.

**Results.:**

We obtained archipelago-specific data on food consumptions by children of different ages and by pregnant and lactating women during pregnancy and breastfeeding and behaviour, including time spent outdoors and type and construction materials of residences.

**Conclusions.:**

This article presents the first detailed information on several key aspects of daily life on French Polynesian archipelagoes during the 1960s–1970s impacting radiation exposure. Important behavior and food consumptions data obtained in this study are being used to improve the radiation dose estimates and to update the risk analysis reported earlier by correcting biases from previous assumptions and by providing better estimates of the parameter values important to radiation dose assessment.

## Introduction

From 1966 to 1974, France conducted 41 atmospheric nuclear tests in French Polynesia, at atolls Mururoa and Fangataufa in the southeast of the Tuamotu Archipelago. In early 2000s, a case-control study based on all alive cases of differentiated thyroid cancer developed between 1985 and 2003 in patients aged less than 25 years at the time of atmospheric nuclear testing was initiated by the French National Institute of Health and Medical Research (INSERM). In this study, which included 602 subjects (so-called Phase I of the study), an increased risk of thyroid cancer was found in the exposed population, despite low levels of radiation dose (de Vathaire et al. 2010). The risk estimate was low and was based on sparse available data for the assessment of the radiation exposure of the subjects. Despite the data limitations, the thyroid doses were estimated for each study subject and for each atmospheric nuclear weapons test that contributed substantially to the local deposition of radionuclides, as described by Drozdovitch et al., (2008). The components of the calculated thyroid doses were those resulting from ^131^I intakes (major source of thyroid exposure), intakes of short-lived radioiodines (^132^I, ^133^I, ^135^I) and ^132^Te, external exposure from gamma-emitted radionuclides deposited on the ground, and ingestion of long-lived ^137^Cs. 

The reconstruction of the individual doses was based on the available information on both the radiation field, which was generalized to the study subjects who resided in the same island or atoll, and individual behaviors that affected both external and internal exposures. The behavioral and dietary parameters necessary for the dose reconstruction could be divided into the following categories: (1) construction materials of residences and schools that define shielding properties of buildings against ionizing radiation, (2) time spent outdoors by children at different ages, (3) consumption patterns of foodstuffs by children at different ages, (4) consumption patterns of foodstuffs by pregnant and lactating women that is important for the subjects exposed in utero and due to intake of ^131^I with breast milk, (5) source of drinking water, and (6) culinary practices that might reduce concentration of radionuclides in cooked product in comparison with raw product. To collect individual data, the study questionnaire was developed and administered to each study subjects by face-to-face personal interview in the early 2000s. This questionnaire included questions about (1) places of residence during atmospheric nuclear testing campaign in 1966‒1974, (2) the consumption of various foodstuffs during early adolescence at age 15 y, (3) source of drinking water, i.e. individual cistern, communal cistern, other, and (4) type of residence, i.e. apartment, house, other. 

In implementation of dose reconstruction for Phase I of study, we faced with the following limitations in using of information collected during the personal interviews:

– Question on construction material of residence was missed, only type of residence was asked. 

– Data on consumption of various foodstuffs only at early adolescence at age 15 y were collected. The consumption at any age less than 15 was then scaled from the consumption at age 15 y using the results of a survey on age-specific diet conducted in Tahiti in 1980-1982 by Grouzelle et al., (1985). However, this survey has two main weaknesses: (1) it was carried out around 10 years after the time of nuclear weapon testing, and (2) it does not include data for islands and atolls other than Tahiti. 

– Questions on consumption of leafy vegetables (cabbage Chinese or ‘pota’ and watercress salad or ‘cresson’) and fâfâ (very similar to spinach), which are one of the most important sources of ^131^I intake with diet, were not included in the questionnaire. 

– Information on some important behavioral and dietary parameters necessary for the dose reconstruction, i.e. consumptions of foodstuffs by pregnant and lactating women and as well as time spent outdoors by children at different ages, was not collected. 

The epidemiological study of thyroid cancer was recently extended to include 348 additional subjects (so-called Phase II of the study). It was decided to improve the thyroid dose estimates and to reduce their uncertainties by the collection of historical data on lifestyle of French Polynesians at the time of nuclear tests. Because four to five decades had elapsed since the nuclear tests were conducted, the focus group discussion and key informant interview methodology was chosen to overcome normal memory recall limitations (McLafferty, 2004). Many of the study subjects were too young at the time of exposure to recall their consumption habits, therefore, mothers and caretakers of children were considered to be a more reliable source of those data. Moreover, mothers of many study subjects were no longer alive, and women, whose children were younger than 21 years during the period of atmospheric testing in 1966‒1974, were selected to participate in focus groups to provide information about their children’s behavior. 

The purposes of this article are to briefly describe the data collection by these methods and to provide the values of the important parameters used for thyroid dose reconstruction. 

## Materials and Methods

*Focus groups*

**Figure 1:**
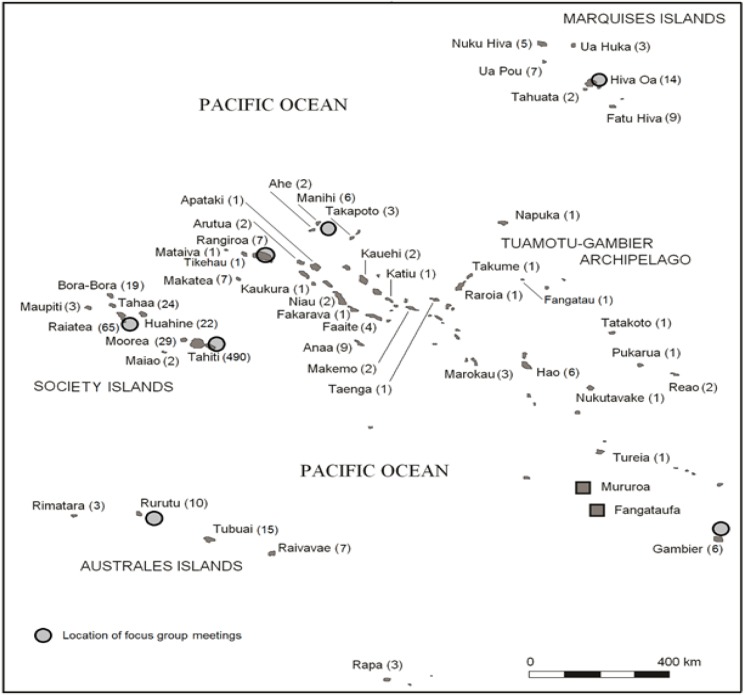
Locations Selected for Focus Group Interviews and Key Informant Interviews (Circles). The numbers of study subjects who resided in each island or atoll are given within parentheses

The focus groups field study was conducted in three phases in August-September 2016, in February, and in May-June 2017 in seven islands and atolls that represent all archipelagoes of French Polynesia (Figure 1). The focus group locations covered the places of residence for 75% of the 950 subjects included in the study. In each island or atoll, two focus-group meetings (except Tahiti where 7 focus-group meetings were conducted) with up to eight women (mothers and caregivers of children living on the island or atoll at the time of the nuclear tests) were conducted. In total, 108 women participated in the focus-group meetings. The age of the women who participated in the focus groups ranged from 57 to 95 y, with a mean and median age of 71 y. The focus-group participants were selected from the population of residents of the fallout-affected islands / atolls at the time of the tests. The women were identified and contacted by the staff of the town halls of the local municipalities.

The topics for discussion in the focus groups were intended to reflect the social practices at the time of the nuclear tests. Women mainly took care of children and, therefore, were a reliable source of information on diet and activity patterns of children. The focus-group participants provided information about the time children spent outdoors daily and about children’s consumption patterns at different ages (0-12 mo, 1-3 y, 4-6 y, 7-14 y, and 15-21 y). We found that only a few participants of the focus groups had children aged 15-21 y at the time of the testing, therefore women were asked about their own consumption habits at age 15-21 y as surrogate data. Data obtained from the mothers for age 15-21 y were combined with data reported for the children of the same age group. According to the study participants, the diet remained constant between 1966 (and even 10 or 15 years earlier) and 1974; newer foods were not introduced into the diets until the late 1970s. Therefore, behavior and dietary information collected from the focus groups reflects the situation during the years of exposure. To capture the variability of lifestyle patterns, at least two groups per archipelago were conducted. 

Participants of the focus groups were also asked about their own food consumptions during pregnancy and breast feeding. This information is important for the dose reconstruction as 96 study subjects (10.1% of the total) were exposed while in utero and 131 study subjects (13.8% of the total) were breastfed in 1966–1974.

During the focus group meeting, to stimulate participant memory, the moderator asked open-ended questions for each topic of discussion. To elicit the information, answers of the participants were writing down on data collection sheets. 

*Key informant interviews*

 To collect information about supplemental factors that are also required for environmental dose reconstruction, individual interviews were conducted with ‘key informants’ in addition to the focus group meetings. Eighteen persons with extensive experience and knowledge of different aspects of daily life in the study area at the period of atmospheric nuclear weapons testing were interviewed in 2016‒2017 in the six islands and atolls. The age of the 8 female and 10 male key informants ranged from 58 to 83 y, with a median age of 72 y. During 1966‒1974, these individuals worked as teachers (n=7), politicians and local authorities (n=5), owners of business (n=2), fishermen (n=2), agriculture workers (n=1), and military personnel (n=1). 

The key informants provided information on lifestyle and dietary practices in the mid-1960s – mid-1970s, including: (1) consumption of fresh cow’s milk and milk products in Tahiti; (2) consumption of ‘exotic’ food on different archipelagoes; (3) the fraction of the families that lived in different types of residences (e.g. family house, multistore house, straw house) and construction materials of residences; (4) culinary practices for leafy vegetables; (5) attendance of schools by children and construction materials of schools; (6) peculiarities of diet for pregnant and lactating women; and (7) sources of drinking water for residents as well as cisterns’ size and area of rain water collection for family and communal cisterns. The oral responses of the key informants were documented on paper forms by interviewer. 

All numerical information accrued from the focus group participants and provided by the key informants was synthesized into tables and databases for further analysis, as presented below. The characteristics of the various groups in terms of demography, consumption, time spent outdoors, etc., are presented according to basic descriptive statistics, such as frequency, arithmetic mean and standard error of mean.

## Results

Two types of data were derived from the focus-groups sessions. First, individual data, i.e. answer from each participant on each question, were collected on (1) the frequency and amounts of fresh milk, leafy vegetables and fâfâ (that are the most important sources of ^131^I intake with diet) consumed by children at different ages (0-12 mo, 1-3 y, 4-6 y, 7-14 y, and 15-21 y), (2) the frequency and amounts of foodstuffs (fresh cow milk, leafy vegetables, fâfâ, coco water, coco milk, uru (fruit of bread tree), banana, mango, papaya, manioc, taro, sweet potatoes, poultry, beef, pork, goat meat, benitier and fish) consumed by women during pregnancy and breast feeding, (3) sources of drinking water, and (4) type of residence and construction material of residence. Second, group consensus data were collected on (1) the frequency and amounts of foodstuffs (see list above, except fresh milk, leafy vegetables, and fâfâ) consumed by children at different ages and (2) time spent outdoors by children at different ages. Individual data were used to determine inter-individual variability between children as well as between pregnant and lactating women while group consensus data were used to estimate age- and archipelago-specific parameters. Individual responses from key informants, which reflected their expert opinion, were used for evaluating the variability in parameters between archipelagoes. Data on construction material of residences and sources of drinking water were collected from both focus groups and from key informants that allowed to understand better typical behaviors and conditions as well as their inter-individual variability. The central estimates of behaviour and food consumption parameters are discussed in the sections below. 

*Parameters of external exposure*

*Time spent outdoors*

**Figure 2:**
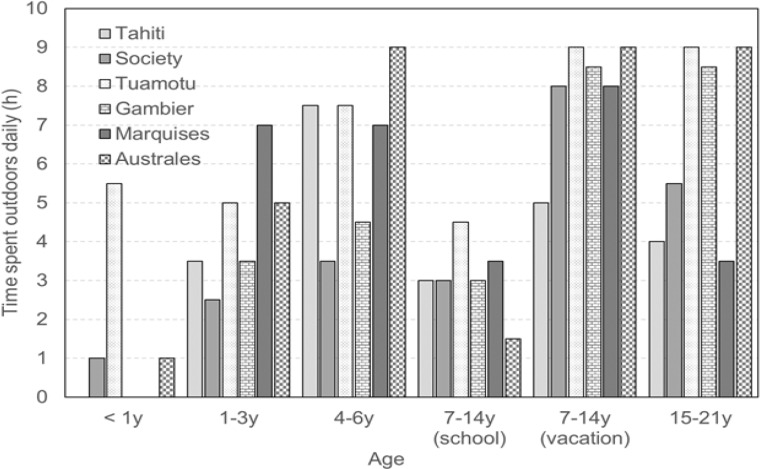
Average Times per Day Spent Outdoors, by Age and Season (only for 7-14 y Children).

More than half of the nuclear tests were conducted during 15 June ‒15 August when school children were on vacation and came back home from boarding schools. Therefore, participants of the study were asked to provide data related to time when school was in session and school was not in session. Figure 2 shows the reported average times per day spent outdoors, by age and season (only for 7-14 y children). For Tahiti island and Marquises islands, the daily time spent outdoors was remarkably similar, except for children aged 1-3 y. In contrast, children, especially young, spent more time outdoors on Tuamotu archipelago compared to other archipelago/islands.

*Construction materials of houses and schools*

**Figure 3:**
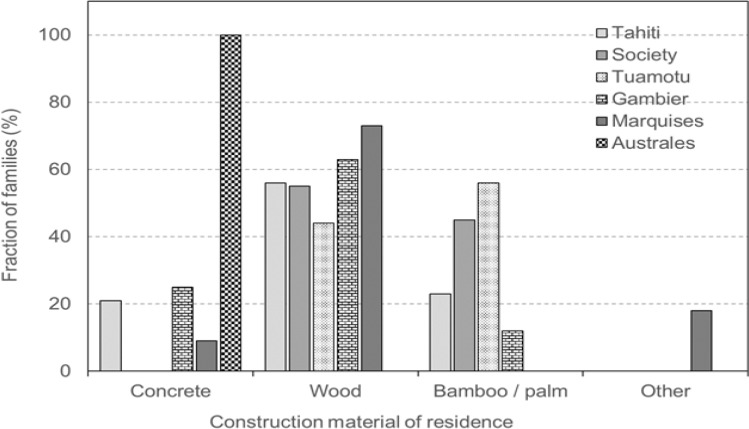
Archipelago (Island)-Specific Fractions of Families that Lived in Houses Build with Different Construction Materials.

Figure 3 shows the archipelago (island)-specific fractions of families that lived in houses build with different construction materials. It should be noted that houses were always in concrete in Australes Islands (specifically in Rurutu), which could be related to cooler climate.

Key informants reported that concrete was practically the only material used to construct schools in all archipelagos in the 1960s–1970s except in Tuamotu archipelago (not shown). In Tuamotu, coral and plywood were used as construction materials for schools along with concrete.

*Parameters of internal exposure*

*Consumption of foodstuffs by children*

Participants in focus groups reported consumption of foodstuffs for 382 children, including 176 females and 206 males. Table 1 presents the fraction of children who consumed fresh cow’s milk, leafy vegetables and fâfâ, which were important sources of ^131^I intake. Consumption of fresh cow’s milk by children was reported only for Tahiti and Raiatea (Society Islands). For a large fraction of children, up to 91%, consumption of locally produced leafy vegetables and fâfâ was reported for all archipelagos, except Tuamotu where fâfâ was not consumed.

Table 2 presents the average archipelago (island)- and age-specific consumption of foodstuffs. Differences between archipelagos were observed for the daily consumption of foodstuffs. The consumption of fâfâ was reported to be highest in the Australes Islands while the lowest consumption was reported in the Gambier. Consumption of fâfâ in the Marquises Islands are based on very few responses as well as in Gambier. As expected, the highest consumption of coco water was reported for Tuamotu archipelago where it supplemented drinking water collected from rainfall. The highest consumption of uru, benitier and fish was also reported for Tuamotu archipelago. Consumption of beef and pork was reported to be rather low (except Marquises Islands). The focus groups at Tuamotu archipelago reported consumption of root vegetables, like manioc, taro and sweet potatoes; however, they were not locally grown. Taro consumed at Gambier was also not locally grown.

*Consumption of foodstuffs by women during pregnancy and lactation*

Table 3 presents consumptions by pregnant and lactating women . In general, there is small difference in fraction of consumers and consumptions between pregnant and lactating women. Fresh cow milk was consumed by 30% of pregnant and breastfed women in Tahiti. Focus group participants reported that a reasonable fraction of women consumed leafy vegetables (up to 100 in Gambier) and fâfâ (up to 89% in Australes Islands) during pregnancy and lactation. 

*Source of drinking water*

Communal and family cisterns were reported to be the only source of drinking water at atolls of Tuamotu archipelago. Use of either communal or family cistern for collection of rain water for drinking purposes was also reported for all other archipelagos (except Gambier) with fraction of 4%, 9%, 36% and 11% reported for Tahiti, Society, Marquises and Australes Islands, respectively. Key informants reported that average size of cisterns was 64,000 L and 11,000 L and area of rain water collection was 200 m^2^ and 30 m^2^ for communal and family cisterns, respectively.

*Culinary practices for leafy vegetables*

Key informants reported that lettuce and watercress salad are consumed as raw products while fâfâ and cabbage Chinese (pota) are boiled or fried with coco milk. 

**Table 1:** Fraction of Consumers (%) of Milk, Leafy Vegetables and Fâfâ among Children of Different Ages

Foodstuff	Archipelago / Island	Age, y				
		<1	1-3	4-6	7-14	15-21
Fresh cow’s milk	Tahiti		6	8	32	21
	Society		DN^1^	DN^1^		
Leafy vegetables	Tahiti		9	35	71	71
	Society	42^2^	50	25	22	53
	Tuamotu			28	64	61
	Gambier		14	38	62	83
	Marquises		37	47	90	69
	Australes		37	91	100	91
Fâfâ	Tahiti		0.9	19	51	80
	Society	67^2^	52	31	44	67
	Gambier			44	35	67
	Marquises		11	11		31
	Australes	22^3^	56	73	67	91

^1^, There was consumption, however, numerical estimation was not provided by the focus group participants;^ 2^, Infants aged 6-12 months; ^3^, Infants aged 8-12 months.

**Table 2:** Daily Consumption^1,2^ (g(mL) d^-1^) of Foodstuffs by Children of Different Ages in mid 1960s - mid 1970s.

Foodstuff	Archipelago / Island	Age, y				
		<1	1-3	4-6	7-14	15-21
Fresh cow’s milk	Tahiti		371±70	321±53	213±25	314±62
	Society		DN	DN		
Leafy vegetables	Tahiti		31±3.7	36±3.2	61±7.9	79±14
	Society	109±34	147±27	*210* ^3^	*7*	121±55
	Tuamotu			*30*	110±32	96±27
	Gambier		*60*	76±5.6	62±14	93±40
	Marquises		5.2±1.4	88±14	*260*	92±31
	Australes		48±7.3	73±17	110±44	86±15
Fâfâ	Tahiti		*2.9*	38±12	54±8.0	61±8.2
	Society	12±1.8	18±1.2	11±1.6	69±18	77±8.8
	Gambier			21±5.9	7.8±1.4	14±3.1
	Marquises		8.5±2.9	*2.7*		61±30
	Australes	104±25	107±13	100±27	130±19	160±32
Coco water	Tahiti	2.5^4^	30	73	70	139
	Society	1	21	59	123	270
	Tuamotu	160	200	342	517	600
	Gambier	100	100	109	141	202
	Marquises	30	23	44	297	297
	Australes			23	46	61
Coco milk	Tahiti	1.5^4^	5.6	36	35	44
	Society	2	30	59	116	123
	Tuamotu		17	80	80	80
	Gambier		11	26	51	60
	Marquises	30	62	62	128	131
	Australes		40	66	100	132
Uru	Tahiti		7.6	73	133	225
	Society	13^4^	37	54	81	156
	Tuamotu		22	178	334	786
	Gambier		41	80	89	136
	Marquises		58	190	197	197
	Australes		30	39	44	53
Banana	Tahiti	7^5^	79	159	190	247
	Society	24^4^	54	94	198	373
	Tuamotu		21	183	342	354
	Gambier		68	133	544	406
	Marquises	38	361	361	367	389
	Australes	90	90	199	212	318
Mango^6^	Tahiti	21	137	186	283	356
	Society	42^4^	103	222	260	574
	Tuamotu^7^			93	112	330
	Gambier		61	272	516	731
	Marquises	19	37	56	223	483
	Australes	78	156	520	728	689
Papaya	Tahiti	26	40	44	45	105
	Society	10^4^	17	29	54	69
	Tuamotu		98	141	266	398
	Gambier		117	242	249	235
	Marquises	5	48	343	355	355
	Australes	16	16	43	75	182
Manioc	Tahiti		40	82	105	154
	Society	4.9	20	38	56	88
	Tuamotu^7^				18	26
	Gambier		41	216	288	378
	Marquises	13	7.8	7.8	10	14
	Australes		131	197	258	581
Taro	Tahiti		26	68	77	101
	Society	7.1^4^	24	45	65	149
	Tuamotu^7^				18	26
	Gambier^7^		41	74	80	107
	Marquises		-	1.5	4.8	8.4
	Australes		122	276	322	351
Sweet potatoes	Tahiti		36	83	95	118
	Society	4.9^4^	20	38	56	149
	Tuamotu^7^			44	105	100
	Gambier			12	18	25
	Marquises			10	10	23
	Australes		26	177	260	441
Poultry	Tahiti		12	53	62	84
	Society	14	14	25	29	40
	Tuamotu		6.3	30	86	76
	Gambier		51	51	77	103
	Marquises		5.1	28	55	57
	Australes	15	20	46	69	82
Beef	Tahiti		1.1	6.9	8.2	8.5
	Society		4.8	7	12	14
	Gambier			0.9	1.4	1.9
	Marquises			2.7	6.1	13
Pork	Tahiti			6.7	9	10
	Society		4.8	9.3	16	18
	Tuamotu			1.6	3.3	7.1
	Gambier		4.8	13	18	25
	Marquises			82	177	179
	Australes	3.5	4.6	4.6	18	28
Goat meat	Marquises			53	117	234
Benitier	Tahiti		1	3.1	15	26
	Society				3.2	8.5
	Tuamotu			12	22	54
	Gambier			2.1	3.1	9.4
	Marquises			14	21	21
Fish	Tahiti	2.1	48	107	190	273
	Society	41^4^	94	170	237	316
	Tuamotu		44	149	843	1020
	Gambier		31	87	108	144
	Marquises		27	108	235	237
	Australes	138^5^	138	299	345	391

^1^, Arithmetic mean ± standard error of mean among children for whom consumption of cow’s milk, leafy vegetables and fâfâ was reported. For other products arithmetic mean between reported focus group consensuses weighted by number of women in focus groups is shown; ^2^, Locally produced food otherwise indicated; ^3^, For values highlighted with Italic, all focus group participants reported the same consumptions by their children; ^4^, Infants aged 6-12 months; ^5^, Infants aged 8-12 months; ^6^, Consumption is given for fruit’s season December−January and July−August; ^7^, Not locally produced.

**Table 3:** Fraction of Consumers (%) and Daily Consumption^1,2^ by Pregnant and Lactating Women in mid-1960s – mid 1970s

Foodstuff	Archipelago / Island	Pregnancy		Lactation	
		Fraction (%)	Consumption rate (g(mL) d^-1^)	Fraction (%)	Consumption rate (g(mL) d^-1^)
Fresh cow’s milk	Tahiti	30	151±22	30	173±22
	Society	30	147±13	30	147±13
Leafy vegetables	Tahiti	72	97±14	70	99±14
	Society	83	70±19	75	68±20
	Tuamotu	40	65±26	40	65±26
	Gambier	100	71±22	100	71±22
	Marquises	44	93±11	44	93±11
	Australes	89	137±43	89	137±43
Fâfâ	Tahiti	66	74±17	66	75±17
	Society	50	59±12	67	55±11
	Gambier	43	145±82	43	145±82
	Australes	89	196±77	89	196±77
Coco water	Tahiti	74	288±57	72	236±47
	Society	67	194±50	58	106±31
	Tuamotu	100	360±66	93	364±52
	Gambier	100	309±49	100	309±49
	Marquises	56	105±37	56	184±61
	Australes	78	156±65	78	156±65
Coco milk	Tahiti	85	77±15	83	68±10
	Society	75	60±14	83	56±13
	Tuamotu	93	99±23	87	105±28
	Gambier	71	56±25	71	56±25
	Marquises	100	311±19	100	302±28
	Australes	78	105±44	78	105±44
Uru	Tahiti	77	197±33	72	204±34
	Society	83	90±22	83	93±24
	Tuamotu	93	469±70	93	460±55
	Gambier	100	213±63	100	213±63
	Marquises	100	90±27	100	66±5
	Australes	78	179±73	78	179±73
Banana	Tahiti	89	227±28	83	224±29
	Society	67	163±46	75	167±41
	Tuamotu	93	175±30	87	181±29
	Gambier	100	437±115	100	437±115
	Marquises	89	448±90	89	373±100
	Australes	100	360±107	100	360±107
Mango^3^	Tahiti	91	408±47	87	403±49
	Society	75	559±99	75	458±83
	Tuamotu^4^	33	572±32	33	572±32
	Gambier	100	334±63	100	334±63
	Marquises	89	613±118	78	591±149
	Australes	67	576±144	67	576±144
Papaya	Tahiti	79	238±37	77	231±38
	Society	67	234±56	75	280±49
	Tuamotu	100	375±68	100	375±68
	Gambier	71	305±123	71	305±123
	Marquises	89	437±86	89	381±90
	Australes	78	295±119	78	295±119
Manioc	Tahiti	60	100±35	64	66±16
	Society	83	74±14	83	74±13
	Gambier	100	133±23	100	133±23
	Marquises	78	102±53	78	102±53
	Australes	89	202±67	89	202±67
Taro	Tahiti	91	129±17	96	122±16
	Society	83	78±14	83	74±13
	Gambier^4^	71	122±27	71	122±27
	Marquises	56	36±12	56	36±12
	Australes	100	345±102	100	345±102
Sweet potatoes	Tahiti	60	63±11	62	61±10
	Society	83	78±14	83	74±13
	Gambier	86	96±33	86	96±33
	Marquises	11	*131* ^5^	11	*131*
	Australes	78	152±67	78	152±67
Poultry	Tahiti	74	70±10	72	72±10
	Society	75	64±16	75	67±15
	Tuamotu	87	68±7.5	93	71±13
	Gambier	100	148±55	100	148±55
	Marquises	78	191±43	78	191±43
	Australes	78	74±16	78	74±16
Beef	Tahiti	51	36±5.9	53	36±5.6
	Society	58	31±7.1	58	27±5.2
	Tuamotu^4^	47	3.1±0.8	47	3.1±0.8
	Gambier	57	44±19	57	44±19
	Marquises	78	55±17	78	55±17
	Australes	33	41±13	33	41±13
Pork	Tahiti	38	49±13	38	49±13
	Society	42	50±20	50	50±16
	Tuamotu	87	52±19	80	56±20
	Gambier	71	69±26	71	69±26
	Marquises	78	160±57	78	160±57
	Australes	56	45±10	56	45±10
Goat meat	Marquises	78	65±27	78	65±27
Benitier	Tahiti	51	45±10	49	39±10
	Society	42	47±15	42	51±14
	Tuamotu	93	53±50	87	54±67
	Gambier	57	31±8.6	57	31±8.6
	Marquises	67	28±11	67	28±11
	Australes	22	58±22	22	58±22
Fish	Tahiti	98	233±22	100	245±22
	Society	83	270±56	83	302±51
	Tuamotu	100	504±49	100	504±49
	Gambier	100	242±68	100	242±68
	Marquises	67	218±80	67	218±80
	Australes	100	289±61	100	289±61

^1^, Arithmetic mean ± standard error of mean among children for whom consumption of cow’s milk, leafy vegetables and fâfâ was reported. For other products arithmetic mean between reported focus group consensuses weighted by number of women in focus groups is shown” with “women who reported consumption of given foodstuff; ^2^, Locally produced food otherwise indicated; ^3^, Consumption is given for fruit’s season December−January and July−August; ^4^, Not locally produced; ^5^, For values highlighted with Italic, all focus group participants reported the same consumptions.

**Figure 4:**
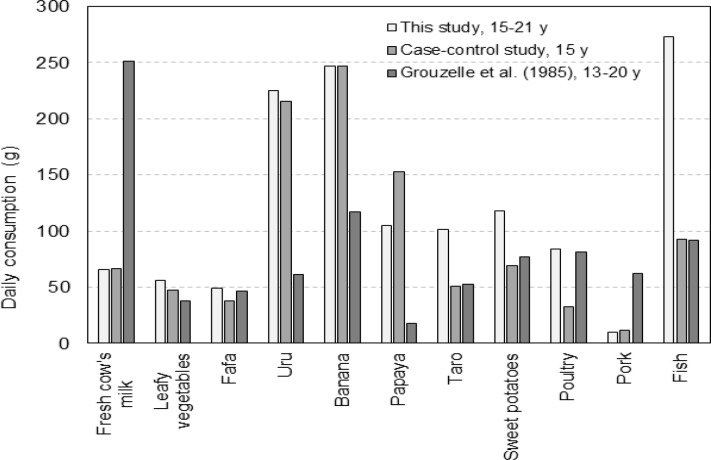
Comparison of Daily Consumption for Tahiti Obtained in Our Study with Those Reported by the Study Subjects of a Case-Control Study and with Literature Data (Grouzelle et al., 1985).

## Discussion

Archipelago differences in diet are clearly seen in the data collected in our study. Consumption of fresh cow milk in the mid-1960s–mid-1970s was reported only for Tahiti and Raiatea. Residents of Tuamotu archipelago did not consume fâfâ at that time, and very few consumers of fâfâ were reported in the Marquises Islands. The previous dose assessment used very general data on diet of Polynesian women during pregnancy and lactation. The archipelago-specific consumption data obtained in our study for these women will substantially improve the estimates of radiation dose to the thyroid gland for persons exposed in utero and during infancy, one of the most exposed age groups of population.

We compared the consumption rates of foodstuffs obtained from three sources for persons aged around 15 y old who resided in Tahiti at the time of testing: our focus group study, reported by 300 subjects of a case-control study and available literature data (Grouzelle et al., 1985) (Figure 4). There is reasonable agreement between the focus groups study and a case-control study on the consumptions of the most important sources of ^131^I intake – fresh cow’s milk, leafy vegetables, fâfâ (for 55 subjects from Phase II of a case-control study) - as well as on the consumptions of uru, banana, papaya and pork. Consumption of cow’s milk (important sources of ^131^I intake) reported by Grouzelle et al., (1985) is much higher than that reported by the focus groups and by the subjects of a case-control study. However, focus groups and the subjects of a case-control study were asked about consumption of locally produced fresh cow’s milk while Grouzelle et al., (1985) did not distinguish between locally produced milk and milk reconstituted from imported powder. 

Ferro-Luzzi (1962) reported per capita daily consumption of 13 g of beef and of 19 g of pork (average for whole territory of French Polynesia), and 172 g of manioc, 156 g of taro and 132 g of banana (average for Tahiti and Moorea). Consumptions of these foodstuffs obtained in our study for young adults (15-21 y) for the same territories are, in general, consistent with those reported by Ferro-Luzzi (1962) for the early 1960s. 

The focus group discussions and key informant interviews are retrospective data collection strategies that provide more reliable recall than individual subject interviews. Low validity and reproducibility of data on recalled individual diet are typically characterized for recollections exceeding 10 years (Willett, 1998) and recall of diet in distant past is strongly influenced by present dietary habits (Rohan and Potter, 1984). Focus group discussion helps to stimulate recall about lifestyle questions and overcome low reproducibility in providing information. Interaction of focus group participants is a unique and compelling feature where participants share their experiences to provide ‘true’ group consensus data as well as the reasons for differences among participants (Kitzinger 1995). However, we observed during the study that individual opinion may be inflected or influenced by group consensus; this may be a limitation of focus group strategy. 

The focus-group methodology was successfully used to collect quantitative and qualitative data on lifestyle and occupational habits for the purposes of retrospective dose reconstruction for radiation epidemiology studies of population exposed in 1949–1962 to fallout from Semipalatinsk nuclear test site in Kazakhstan population (Drozdovitch et al., 2011; Schwerin et al., 2010) and nuclear medicine technologists who diagnostic radioisotope procedures in the 1950s–mid 1970s (Drozdovitch et al., 2014).

Using the focus group and key informant interviews we collected information on events in distant past (about 50 years ago) from residents of islands and atolls who had not migrated elsewhere. The use of focus group participants as surrogates to the study subjects is justified because during the 1960s–1970s life in French Polynesia could be described as uniform within archipelagoes due to limited food variety.

This article presents the first detailed information on several key aspects of daily life on French Polynesian archipelagoes during the 1960s–1970s. The information obtained in this study is being used to improve the dose estimates and to update the risk analysis reported by de Vathaire et al., (2010) by correcting biases from previous assumptions and by providing a better assessment of the parameter values important to radiation dose estimation. 
